# Dysregulation of Gene Expression in a Lysosomal Storage Disease Varies between Brain Regions Implicating Unexpected Mechanisms of Neuropathology

**DOI:** 10.1371/journal.pone.0032419

**Published:** 2012-03-05

**Authors:** Michael K. Parente, Ramona Rozen, Cassia N. Cearley, John H. Wolfe

**Affiliations:** 1 Research Institute of the Children's Hospital of Philadelphia, Philadelphia, Pennsylvania, United States of America; 2 W.F. Goodman Center for Comparative Medical Genetics, School of Veterinary Medicine, University of Pennsylvania, Philadelphia, Pennsylvania, United States of America; Washington University, United States of America

## Abstract

The characteristic neurological feature of many neurogenetic diseases is intellectual disability. Although specific neuropathological features have been described, the mechanisms by which specific gene defects lead to cognitive impairment remain obscure. To gain insight into abnormal functions occurring secondary to a single gene defect, whole transcriptome analysis was used to identify molecular and cellular pathways that are dysregulated in the brain in a mouse model of a lysosomal storage disorder (LSD) (mucopolysaccharidosis [MPS] VII). We assayed multiple anatomical regions separately, in a large cohort of normal and diseased mice, which greatly increased the number of significant changes that could be detected compared to past studies in LSD models. We found that patterns of aberrant gene expression and involvement of multiple molecular and cellular systems varied significantly between brain regions. A number of changes revealed unexpected system and process alterations, such as up-regulation of the immune system with few inflammatory changes (a significant difference from the closely related MPS IIIb model), down-regulation of major oligodendrocyte genes even though white matter changes are not a feature histopathologically, and a plethora of developmental gene changes. The involvement of multiple neural systems indicates that the mechanisms of neuropathology in this type of disease are much broader than previously appreciated. In addition, the variation in gene dysregulation between brain regions indicates that different neuropathologic mechanisms may predominate within different regions of a diseased brain caused by a single gene mutation.

## Introduction

Intellectual disability is a prominent feature of many monogenic diseases affecting the brain. However, the mechanisms by which specific gene defects lead to cognitive impairment remain obscure. Detailed understanding of the relationship of specific pathologic changes to systems defects is lacking, especially since pathological lesions are often present in many areas of the brain. Little data is available on the effects of global disease on different sub-regions of the brain. To gain insight into regional differences in abnormal functions occurring secondary to a single gene defect, we used whole transcriptome analysis to identify molecular and cellular pathways that are dysregulated in the brain in a mouse model of a lysosomal storage disorder (LSD).

The LSDs constitute a large group of neurogenetic diseases in which un-degraded macromolecules accumulate in the lysosome. Specific neuropathologic features occur in many LSDs, including meganeurites, neurite sprouting, ectopic dendrites, and axonal spheroids [1] [2]. Storage lesions are present throughout the brain, but structural abnormalities can be concentrated in types of neurons or specific regions; e.g. GABAergic neurons exhibit neuroaxonal dystrophy more than other cell types [1] and neurodegeneration can occur in selective regions involved in cognition [3]. Like most neurodegenerative diseases, astrogliosis and neuroinflammation are present in LSD brains [4]. Despite the availability of many well-characterized animal models of LSDs, understanding of the role of cellular and molecular changes in the phenotype of the brain disease is at best incomplete [5], [6].

We investigated the widely studied mouse model of mucopolysaccharidosis (MPS) VII, caused by deficiency of β-glucuronidase (GUSB) [7]. Humans with MPS VII have a broad spectrum of clinical signs including variable intellectual disability [8]. MPS VII mice have widespread storage lesions in the CNS [9]. Gene expression studies on the brain in this and other MPS and LSD models have been limited, however, by using whole brain, large pieces that included parts from multiple sub-regions, pooled samples, small numbers of samples, single substructures, or analysis of a limited number of genes.

Our data show that patterns of aberrant gene expression due to disease vary significantly between major brain sub-structures and involve multiple neural pathways, demonstrating greater complexity of abnormalities than seen previously. 1) Comparing MPS VII to the closely related MPS IIIB (the most extensively studied MPS model) showed a different pattern of inflammatory and immune system activation, reflecting differences in the predominant glycosaminoglycan substrate accumulated in each disease. 2) There was substantial down-regulation of oligodendrocyte genes even though white matter disease is not a feature by histopathology. 3) Many developmental genes were altered even though the brains were from adults with advanced disease. The involvement of many important neural systems, and the variation in which systems are dysregulated between regions, indicate that the mechanisms of neuropathology in this type of disease are much broader than previously appreciated.

## Results

### 1. Variation between regions in normal mouse brain

**Figure 1:**
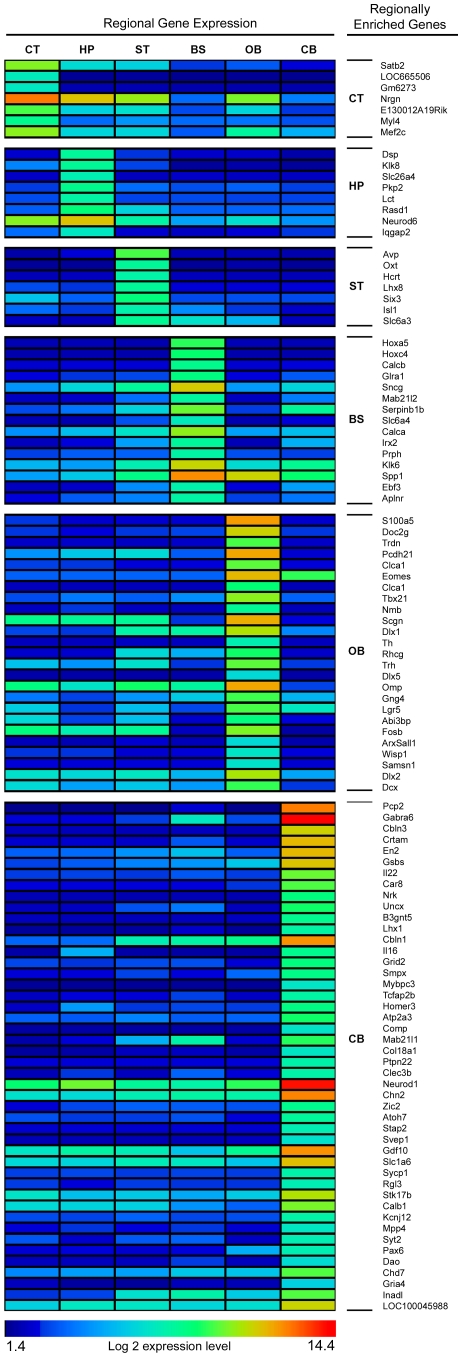
Regional variation of highest expressed genes in normal mouse brain. The log 2 expression levels for genes with a >20 fold increase in expression level over the mean of the other 5 regions were determined and grouped according to highest expressing region. Color bar at bottom shows range of values. Specific genes are listed in right hand column.

Differences between the six regions in the normal mice were analyzed by determining if the region with the maximum expression was significantly above the mean of the other regions at a p* of ≤0.01. The number of genes enriched relative to other regions were: cerebral cortex (CT) 1383 genes (1668 probes); hippocampus (HP) 2165 genes (2656 probes); olfactory bulb (OB) 1877 genes (2341 probes); brain stem (BS) 2923 genes (3718 probes); cerebellum (CB) 1178 genes (1418 probes); rest (largely striatum and thalamus)(ST) 1613 genes (1852 probes). The most highly expressed genes (defined as ≥20 fold change over the mean of the other regions) are shown in a heat map (Figure 1). This list was compared to 22 previously reported regional markers [10], [11], [12], [13], [14], [15]. Twenty (20) of these had the highest expression in the same region in our cohort and the other 2 were found in the region with the second highest level of expression.

### 2. General observations on MPS VII versus normal brains

The large cohort of control and diseased brains allowed analysis of the gene expression changes at the p*≤0.01 level. Since a tightly regulated gene with a small proportional change in magnitude may be biologically significant, a minimum fold change was not used [16]. Overall there were 853 genes (970 probes) altered in at least one region of the mutant mouse brain (Table 1) with the largest number of changes in the olfactory bulb. A number of genes showed significant changes in multiple regions (shared) but the majority were changed only in one brain region (unshared) (Table 2).

**Table 1:** Regional gene changes in MPS VII.

Region	Number of genes (probes) altered
Cortex (CT)	236 (271)
Hippocampus (HP)	180 (214)
Olfactory bulb (OB)	454 (515)
Brain stem (BS)	302 (342)
Cerebellum (CB)	191 (213)
Striatum/Thalmus (ST)	191 (214)

**Table 2:** Number of gene expression changes shared by different regions.

Number of shared regions	Number of genes
All 6	38
5	41
4	40
3	62
2	139
unshared (single region)	591

The significantly changed genes were analyzed using GeneTrail (http://genetrail.bioinf.uni-sb.de) [17] for Gene Ontology (GO) terms [18] and KEGG pathway enrichment [19], and then were evaluated by DAVID (http://david.abcc.ncifcrf.gov/) for functional clustering [20]. The GeneTrail results showed 712 GO-term associations and enrichment of 10 KEGG pathways, with the most significantly over-represented GO-terms shown in Table 3 (full data at http://repository.upenn.edu/wfg_ccmg) and all of the KEGG pathways shown in Table 4. The DAVID analysis showed many related clusters (Table S1), which can be thematically grouped into the following: apoptosis, cell cycle, cell migration, circadian rhythm, development, extracellular matrix/adhesion, immune and inflammatory, ion transport/channels, metabolism, myelination, neural disease, neuron, neuropeptide/hormone, nucleus/gene regulation, signaling, transporter, and ubiquitin.

**Table 3:** Most significantly (p<0.000001) over-represented Gene Ontology terms.

Gene Ontology	expected	observed	p-value(fdr)
lytic vacuole	7.46	40	6.36E-16
lysosome	7.46	40	6.36E-16
immune system process	35.00	91	1.48E-14
vacuole	8.51	40	5.97E-14
positive regulation of biological process	54.61	117	5.05E-13
extracellular region	69.92	137	1.52E-12
response to stimulus	96.89	169	1.95E-11
immune response	20.83	60	2.38E-11
positive regulation of cellular process	48.25	102	5.56E-11
cell proliferation	27.02	69	1.03E-10
positive regulation of immune system process	8.68	35	2.13E-10
protein binding	229.75	319	3.08E-10
regulation of cell proliferation	19.74	54	2.25E-09
response to external stimulus	25.05	62	4.78E-09
ensheathment of neurons	1.49	14	5.75E-09
axon ensheathment	1.49	14	5.75E-09
regulation of immune response	7.94	31	6.83E-09
positive regulation of response to stimulus	7.15	29	1.05E-08
positive regulation of immune response	5.83	26	1.08E-08
regulation of immune system process	12.90	40	1.72E-08
regulation of action potential in neuron	1.67	14	2.70E-08
myelination	1.40	13	2.70E-08
developmental process	126.76	192	4.64E-08
pattern binding	5.00	23	5.31E-08
polysaccharide binding	5.00	23	5.31E-08
response to stress	47.94	92	7.33E-08
regulation of response to stimulus	12.59	38	8.43E-08
cell adhesion	25.48	59	1.09E-07
biological adhesion	25.48	59	1.09E-07
defense response	19.83	50	1.14E-07
cell surface	10.66	34	1.33E-07
immune effector process	7.68	28	1.68E-07
inflammatory response	10.26	33	1.78E-07
lymphocyte mediated immunity	5.44	23	2.29E-07
regulation of action potential	2.02	14	3.08E-07
response to wounding	15.04	41	3.08E-07
carbohydrate binding	13.29	38	3.08E-07
leukocyte mediated immunity	6.01	24	3.14E-07
regulation of multicellular organismal process	30.09	64	4.90E-07
adaptive immune response	5.31	22	6.06E-07
adaptive immune response based on somatic recombination of immune receptors built from immunoglobulin superfamily domains	5.31	22	6.06E-07
regulation of developmental process	39.43	77	6.06E-07
anatomical structure development	92.28	145	6.49E-07
chemical homeostasis	13.82	38	7.31E-07
external side of plasma membrane	7.37	26	9.12E-07

**Table 4:** All KEGG pathways of significantly changed genes (from Genetrail).

KEGG pathway	expected	observed	p-value(fdr)
Lysosome	6.40	28	1.97E-09
Complement and coagulation cascade	3.97	13	8.67E-03
Cell adhesion molecules (CAMs)	6.40	28	3.43E-02
Huntington's disease	3.97	13	3.43E-02
Other glycan degradation	8.78	19	3.43E-02
Adipocytokine signaling pathway	3.70	10	4.71E-02
Cytokine-cytokine receptor interaction	13.32	24	4.71E-02
Glycosaminoglycan degradation	1.06	5	4.71E-02
Pathways in cancer	18.29	31	4.71E-02

**Figure 2:**
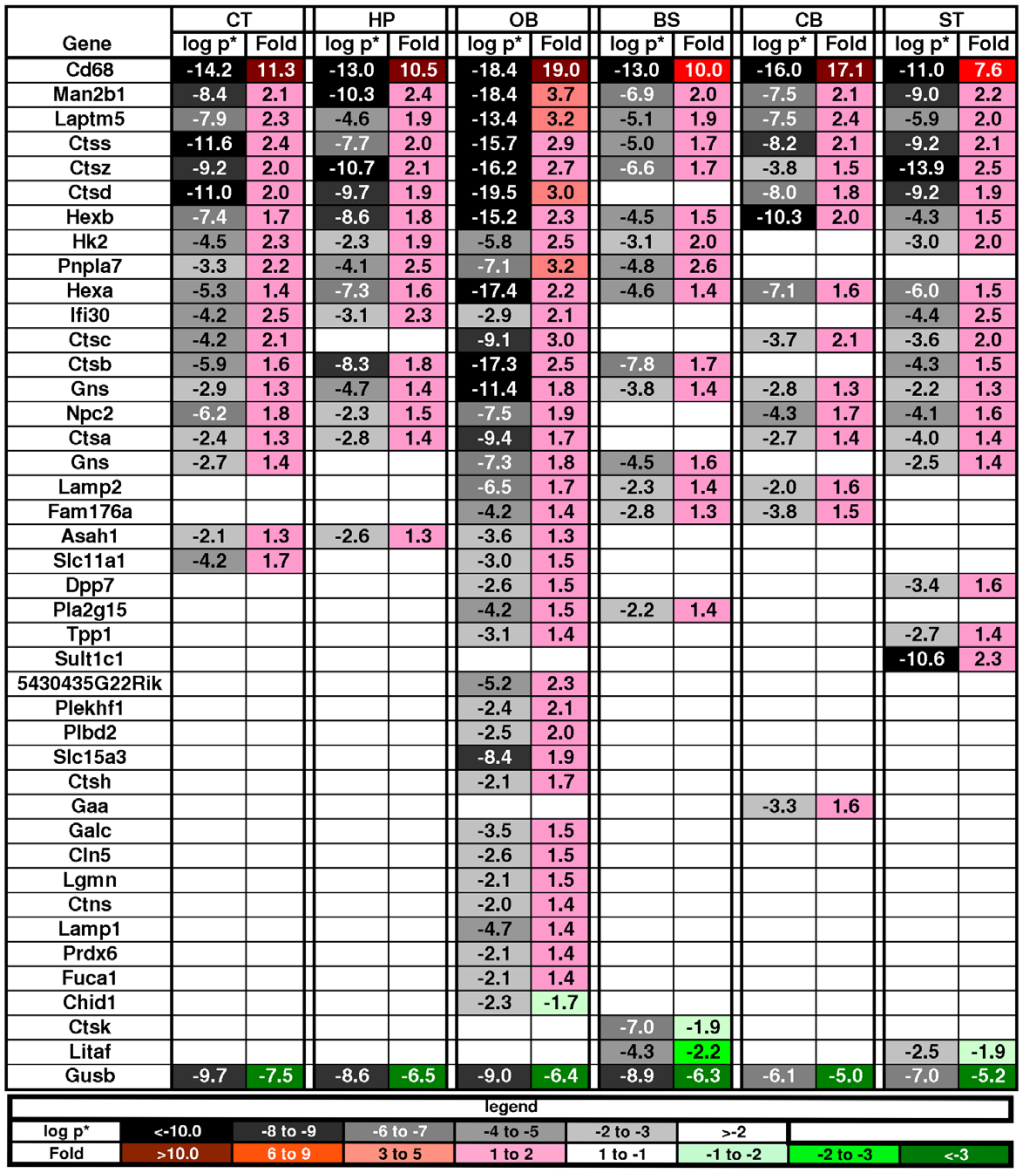
Changes in lysosome genes across regions in MPS VII brain. The key across the bottom shows the magnitude of significance coded in shades of gray and the fold change and direction coded in shades of red for increased expression and shades of green for decreased expression (the same key is used in figures 3–8). The values for each gene in each region are in the boxes. Gusb was down-regulated in all regions, as expected, while other normal lysosomal genes were up-regulated consistent with enzymatic activity data.

Expression of the Gusb gene was significantly down-regulated in all brain regions in the MPS VII mice (average p* = 1.4×10^−7^). This was expected because the mutation is at the 3′ end of the gene and results in a very low level of transcription due to instability of the mutant mRNA [21]. Many other lysosomal genes were up-regulated (Figure 2), which was also expected since secondary elevation of enzymatic activities of other lysosomal enzymes is a common feature in LSDs, including MPS VII [22], [23], [24]. The reduced expression in Gusb and increased expression of Hexb were confirmed by RT-PCR (Fig. S1). In addition to the genes that are listed in the DAVID Lysosome cluster, 5 more recently identified lysosomal genes were also up-regulated [25].

We also examined genes of the recently identified CLEAR (Coordinated Lysosomal Expression and Regulation) network regulated by transcription factor EB (TFEB) [26], [27]. However, the changes in the MPS VII brain were limited to several genes in the lysosomal-hydrolases-and-accessory-proteins group and only one of the autophagy target genes (HIF1A) was changed. None of the TFEB target genes involving lysosomal membranes, acidification, or biogenesis were changed. This suggests that the gene expression changes in a single lysosomal enzyme deficiency state are either unrelated to the CLEAR network or possibly involve other unknown regulatory factors.

### 3. Regional differences between normal and MPS VII mouse brain

Many genes showed changes in various regional combinations. A large number were changed in one region exclusively or lacked change in one region exclusively (Table S2). The direction of change was always the same across regions, with only one exception (Enpp2), which had mixed up/down regulation. For the changes that were found in only one region, the OB had the most (239) followed by the BS (154), CB (96), CT (50), HP (33) and ST (33). Examples are the 11-fold down-regulation in the OB of olfactory marker protein (Omp), expressed specifically in olfactory neurons, which when inactivated alters odorant perception [14], [28]; and the 8-fold down-regulation in the HP of Otx2 which controls neuron subtype identity, the fate of GABAergic neurons, and forebrain development [29], [30], [31]. There were 41 genes that were changed in five of the six regions (unchanged in only one), with the CB having 4 times as many of the unchanged genes as the next closest region (BS). The CT, HP, and OB each had only a single gene that was exclusively unchanged in each region.

There also were regional variations in the clusters of genes related to specific pathology and functions (GO-term categories). For example, the OB ranked highest in immune system process, ensheathment of neurons, inflammatory response, and regulation of action potential, while the CB was the only region with no changes in ensheathment of neurons or action potential. The GeneTrail analysis of each set of regionally unique gene changes found enrichment of GO terms specific to each region. However, most of the functional categories of the DAVID clusters were also identified by the overall analysis, as shown below.

### 4. Gene expression changes underlying specific neuropathologies

The Pathognomonic lesion in MPS VII and the other LSDs is engorged lysosomes in cells [9]; and a number of neuronal dysmorphologies are found in the brain [1]. A number of other morphological, biochemical, and behavioral abnormalities have been found in the MPS VII model and changes in gene expression in specific pathways were found that implicate specific cellular processes that underlie them.

#### 4a. Neurodegeneration

The MPS VII mouse brain has specific regional changes in neurodegeneration, which are concentrated in the hippocampus, cerebral cortex and striatum [3]. The neurodegeneration is characterized by progressive accumulation of ubiquitin and neurofilament and the absence of other neuro-degeneration sub-types characterized by PHF-Tau, alpha-synuclein, or apoptosis [3]. Abnormal levels of ubiquitin and neurofilament are seen in some glia as well as neurons. However, the evaluation of brain homogenates by gene array measures gene expression from a mixture of neural cell types. To determine if alterations in the MPS VII brain regions were associated with specific neural cell types, we compared our results to a panel of genes that are highly expressed in specific isolated populations of neurons, oligodendrocytes, or astrocytes in vitro [32]. Despite the morphologic change in most neurons [9] only 3 out of 80 neuron-specific marker genes were altered: upregulation of Asph in BS, and Cyb561 and Lpl in CB.

Among all the neuronal receptor genes represented in the array, changes were seen in only 4 out of 121, and only in one region each: 1) up-regulation in the BS of the gamma-aminobutyric acid (GABA)-A receptor subunit alpha 6 (Gabra6); 2) down-regulation in the CT of the adrenergic receptor Adra1b; 3) down-regulation in the CT of the class III glutamate receptor, Grm8; and 4) up-regulation in the BS of Slc1a6, the high affinity glutamate transporter (Table S3). There could, though, be significant changes in individual neurons or small nuclei that would not appear against the background of the regional homogenates.

The gene expression arrays scored very high in the order of cluster-related GO-terms for genes associated with apoptosis. In contrast, histopathological analysis shows no evidence for increased apoptosis in the MPS VII mouse brain [3]. Although the GO-term association was high, the gene arrays showed both increases and decreases in expression of apoptotic genes, and for both positive and negative regulators of apoptosis (Table S4) in contrast to a qPCR study [33]. Taken together the data indicates that the net biological effect of the dys-regulated genes in apoptotic pathways was neutral.

There were no significant gene changes for PHF-Tau or alpha-synuclein, consistent with the previous negative histopathology findings [3]. However, there also were no changes detected in neurofilament genes and relatively minor changes in genes associated with ubiquitination, both of which are significantly increased in specific brain regions by immunohistochemical analysis [3]. In the immunohistochemical analysis the numbers of Ubq/NFL+ cells were significantly increased in MPS VII but were a relatively small proportion of each region, thus gene expression changes within these cells would probably not be distinguishable from background in the array analysis since whole region homogenates were assayed.

Changes in expression of other genes that have been associated with neurodegeneration include: 1) cerebellar degeneration-related 2 (Cdr2) which was down-regulated in the HP (although not in the CB); 2) reduced expression in transcription factors associated with neurogenetic diseases (e.g. Fos, JunB) was seen in the CB; and 3) decreased expression of glycine amidinotransferase (Gatm) in the BS, a gene associated with mental retardation [34]. Overall, the data are consistent with histological studies in which neuronal loss is not a major feature of lysosomal storage diseases [1].

#### 4b. Astrogliosis

Astrogliosis is a feature of many neurodegenerative diseases [35], including the MPS VII mouse [3]. Of the top 40 astrocyte cell-specific genes identified in vitro [32], 13 were changed, all of which were up-regulated (Table S5). Genes associated with astrocytosis were up-regulated, including: GFAP, which was up-regulated in all regions and confirmed by RT-PCR (Fig. S1); vimentin, a part of the astrocyte intermediate filament and a hallmark of reactive gliosis [36], in the CT, OB and ST; and markers associated with cell metabolism (Aldoc), cell transport and ion binding (Aqp4, Slc14a1), and signal transduction (Mertk). The most specific marker gene for astrocytes in vitro [32], Aldh1L1, was not changed in any region in the MPS VII brain. Likewise, a number of markers associated with severe astrogliosis were not changed [35], [37]. These data are consistent with the histological findings which show moderate astrogliosis [3].

#### 4c. Microglia, immune system and inflammatory changes

**Figure 3:**
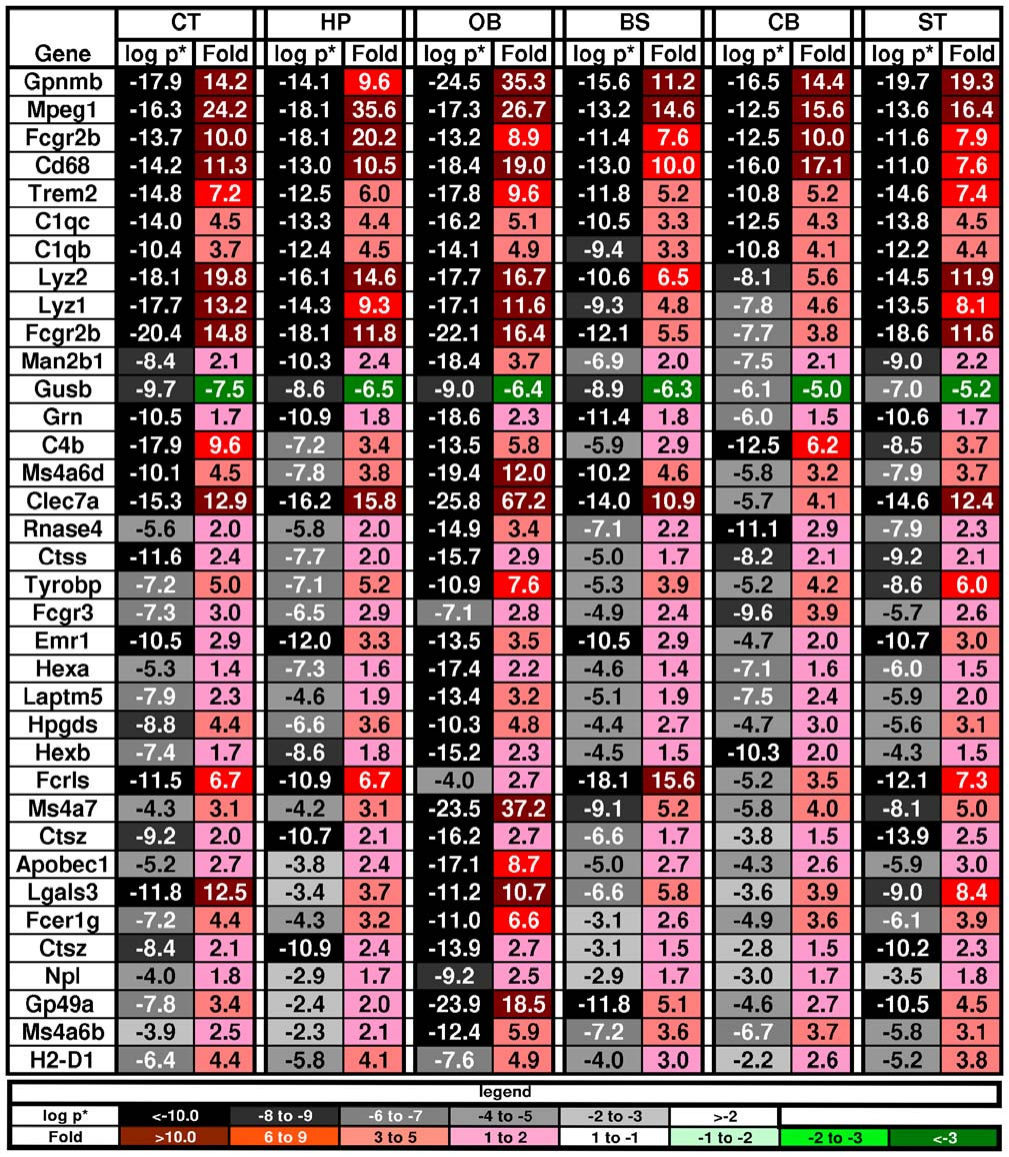
Genes most significantly altered in MPS VII brain. The genes are listed in order of decreasing average p* (step-up p value) across regions. Only genes with p*<0.0001 are shown. The key is the same as in Fig. 2.

Many of the genes involved in activation of microglia and immunity were among the most significantly up-regulated of all the changed genes in the MPS VII mouse brain, including 25 of the 36 most significant genes (average p* across regions <0.0001) (Figure 3). Some examples were: Gpnmb, a negative regulator of proinflammatory response; Cd68; Fcgr2b, the low affinity Fc receptor for IgG; Clec7a, a receptor which recognizes some glucans from fungi and plants; Mpeg1, a macrophage expressed gene; 3 complement components, C1qc, C1qb, and C4b; Trem2 which may have a role in chronic inflammation; lysozyme 1 and 2; Fcrls, the Fc receptor-like S, a scavenger receptor; and, Hpgds, which plays a role in the production of prostanoids in the immune system. Regional differences were seen, with the largest number altered in the OB.

**Figure 4:**
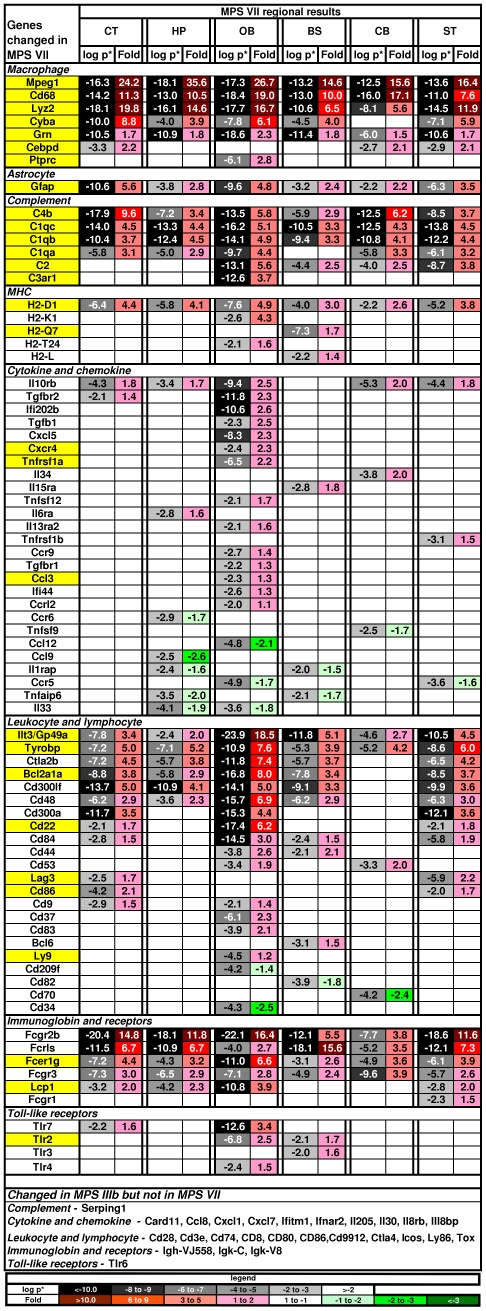
Changes in genes associated with immune system and inflammation. The genes are grouped by class (as used in DiRosario et al., 2009) and then sorted by fold within class. Genes highlighted in yellow were changed in both MPS VII and MPS IIIb [58], [60]. Genes listed at the bottom were changed in MPS IIIb but not in MPS VII. Key as in Fig. 2.

The DAVID analysis found 345 gene changes in 29 functional categories (out of 253) involving the immune system or inflammation (Table S1). These inflammatory categories were in the top one-eighth of the overall list ranked by the strength of the associations. Prominent categorical changes included up-regulation of the cathepsin genes, the products of which are released by activated microglia, have been implicated in the pro-inflammatory response, and are associated with neuronal death and apoptosis. The cathepsins Ctsa, Ctsb, Ctsc, Ctsd, Ctsh, Ctss and Ctsz were highly up-regulated in all brain regions, while Ctsk was only changed in the BS and was down-regulated. The toll-like receptors (TLRs) play a role in innate immunity and TLRs 2, 3, 4 and 7 were up-regulated in some regions (Figure 4). There were also a number of changes in the complement pathway.

However, many other genes that associated with a pro-inflammatory response were not changed in the MPS VII brains. Interleukins are the primary cytokines seen in inflammation, but only a few of these were changed (Figure 4). The biggest change was in the interleukin-10 receptor beta, which was up-regulated in 5 regions and is involved in an anti-inflammatory pathway, but IL10 itself was unchanged. The major pro-inflammatory cytokine, IL1β, was also unchanged while the IL1 receptor accessory gene (IL1rap), which is a necessary part of the interleukin 1 receptor complex, was down-regulated in two regions. The pleiotrophic IL6 was unchanged, but Stat3, the anti-apoptotic effector of IL6, was up-regulated in the OB. Chemokine changes were also largely absent with the exception of the up-regulation of Ccl3 and the down-regulation of Ccl12 in the OB and the down-regulation of Ccl9 in the CT. Another important set of pathways in inflammation involves the mitogen-activated protein kinase (MAPK) cascades [38] but none of the Mapk genes were altered. Additionally, the genes for the GO-term “Leukocyte transendothelial migration” were under-represented in the GeneTrail analysis from our list of changed genes. Finally, several anti-inflammatory molecules were highly up-regulated, including Ilt3/Gp49a, FcγrIIb, Tyrobp, Cd300a and Cd300lf.

#### 4d. Circadian Rhythm

**Figure 5:**
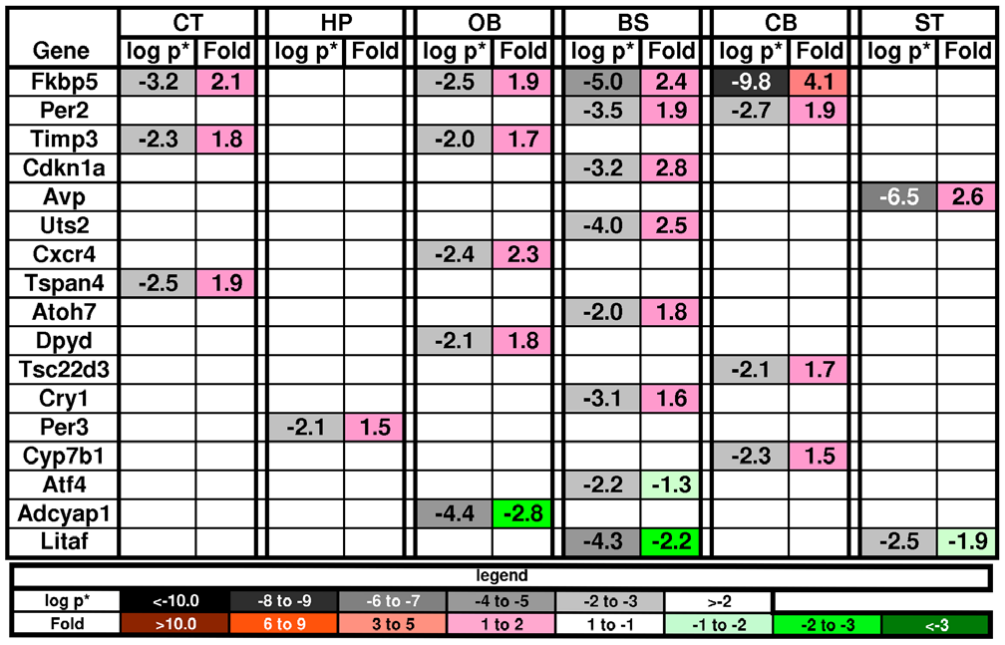
Changes in genes associated with circadian rhythm. The GeneTrail analysis found the GO term circadian rhythm to only be associated with the brainstem. Key as in Fig. 2.

Alterations in circadian rhythm have been documented in the MPS VII mouse [39], and 17 genes known to be involved in circadian rhythm [40] were altered in the MPS VII brain (Figure 5). Eight of these were found in the BS, consistent with the biology of circadian rhythm. Urotensin II, (Uts2), up-regulated in the BS is vasoactive and also has an effect on cholinergic neurons involved in REM sleep [41], [42], [43]. Another vasoactive gene involved in circadian rhythm, arginine vasopressin (Avp), was also up-regulated in the ST. In the GeneTrail analysis the BS was the only one of the 6 regions that generated GO term enrichment related to circadian rhythm and regulation of heart rate.

### 5. Alterations in other neurological systems indicate the presence of important additional pathological processes

In addition to the gene changes associated with previous biochemical, histopathological and behavioral abnormalities, several prominent groupings of gene changes were found that implicate other pathways of pathology.

#### 5a. Gene changes associated with development

**Figure 6:**
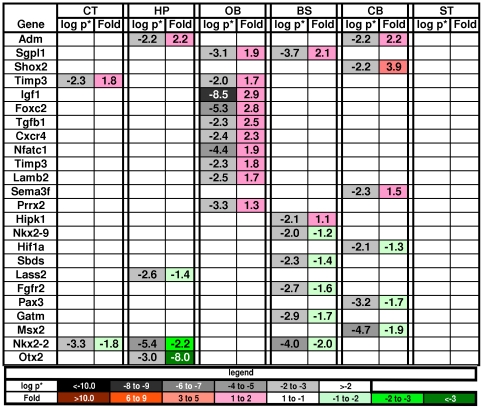
Changes in genes associated with embryonic development and stem cells. Key as in Fig. 2.

**Figure 7:**
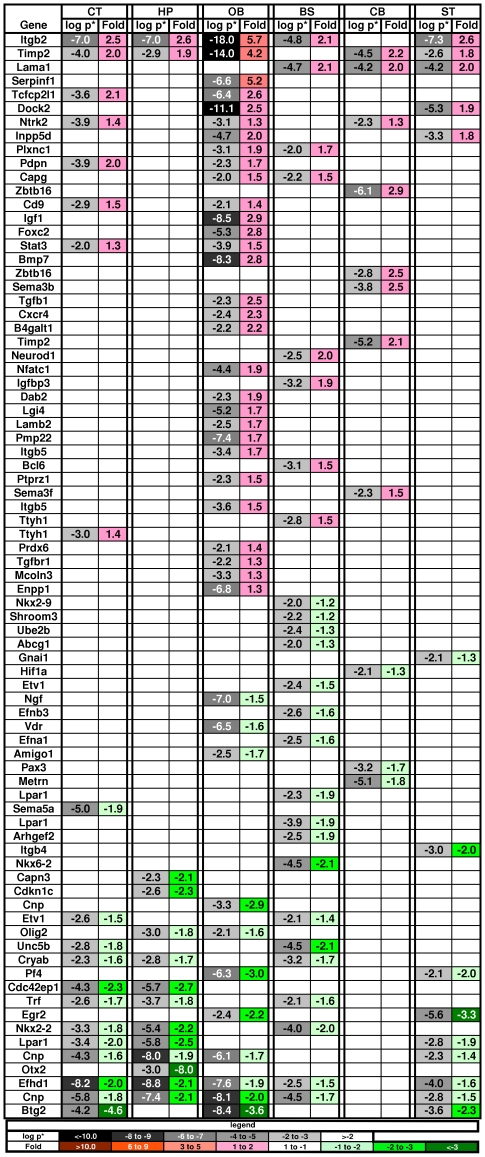
Changes in genes associated with development of neurons. Key as in Fig. 2.

Ten clusters of DAVID pathways were associated with nervous system development terms and included: 1) 4 clusters related to stem cells and other basic development terms such as regeneration, mesenchymal cell and neural crest differentiation, pattern formation, and homeobox genes (Figure 6); 2) 5 clusters related to neuron development, such as neurogenesis, neuron fate commitment, neuron projection development, axonogenesis, and axon guidance (Figure 7); and 3) 1 cluster related to gliogenesis (see section below on myelination).

Specific changes in the MPS VII brain included genes encoding semiphorins, Trk receptors, BMPs, IGF-1, NGF, TGFß, and receptors for FGF2 but not for EGF. Specific developmental genes were increased or decreased across brain regions, but overall there was no dominant pattern of up or down regulation (Figure 7). Increased expression was seen in genes associated with neuronal cell development and differentiation (e.g. Timp2, Adm, Ahnak, Cxcr4, Igf1), growth of neurites or axons (Ntrk2), differentiation of progenitor cells (Stat3), and brain or CNS development (e.g. Tgfbr2, Sepp1, B2m, Cebpa, Cebpd). Down-regulation in the diseased brain was seen in genes associated with quantity of neurites or axons (A2m) and with CNS development (e.g. BTG2, Egr2, Sept4, Sema3b). The largest down-regulation was in the Otx2 (orthodenticle homeobox 2) gene (8-fold with p* = 10^−3^), but only in the HP. This gene encodes a transcription factor (homeobox 2) that controls neuron subtype identity, the fate of GABAergic neurons, and forebrain development [29], [30], [31].

Many other developmental genes that were changed in the MPS brain were associated with development of non-neuronal tissues. For example, skeletal development clusters were seen in both the DAVID and GeneTrail analyses of the brain tissue and skeletal dys-genesis is a major feature of MPS diseases. MPS VII is a disease with a distinct skeletal phenotype with thickened dense bones similar to those seen in osteopetrosis. One of the most significantly changed genes in the MPS VII brain was Gpnmb (osteoactivin), which was originally cloned from an osteopetrosis rat [44], [45]. It is expressed in several areas of the normal adult brain [15] and was very strongly up-regulated in all regions of the MPS VII brain, yet the gene is not included in any of the DAVID clusters for nervous system development. Osteopetrosis is also associated with a decrease in Car2 (carbonic anhydrase 2), which was down-regulated in 5 of 6 MPS VII brain regions. In addition to Gpnmb and Car2, 23 other genes involved in skeleton and bone development were altered in the diseased brain. Developmental genes that were dys-regulated in the MPS VII brain were also associated with development of blood vessels, the urogenital system, endocrine pancreas, striated muscle, exocrine glands, heart, reproductive organs, and retinal photoreceptors.

#### 5b. Myelination

**Figure 8:**
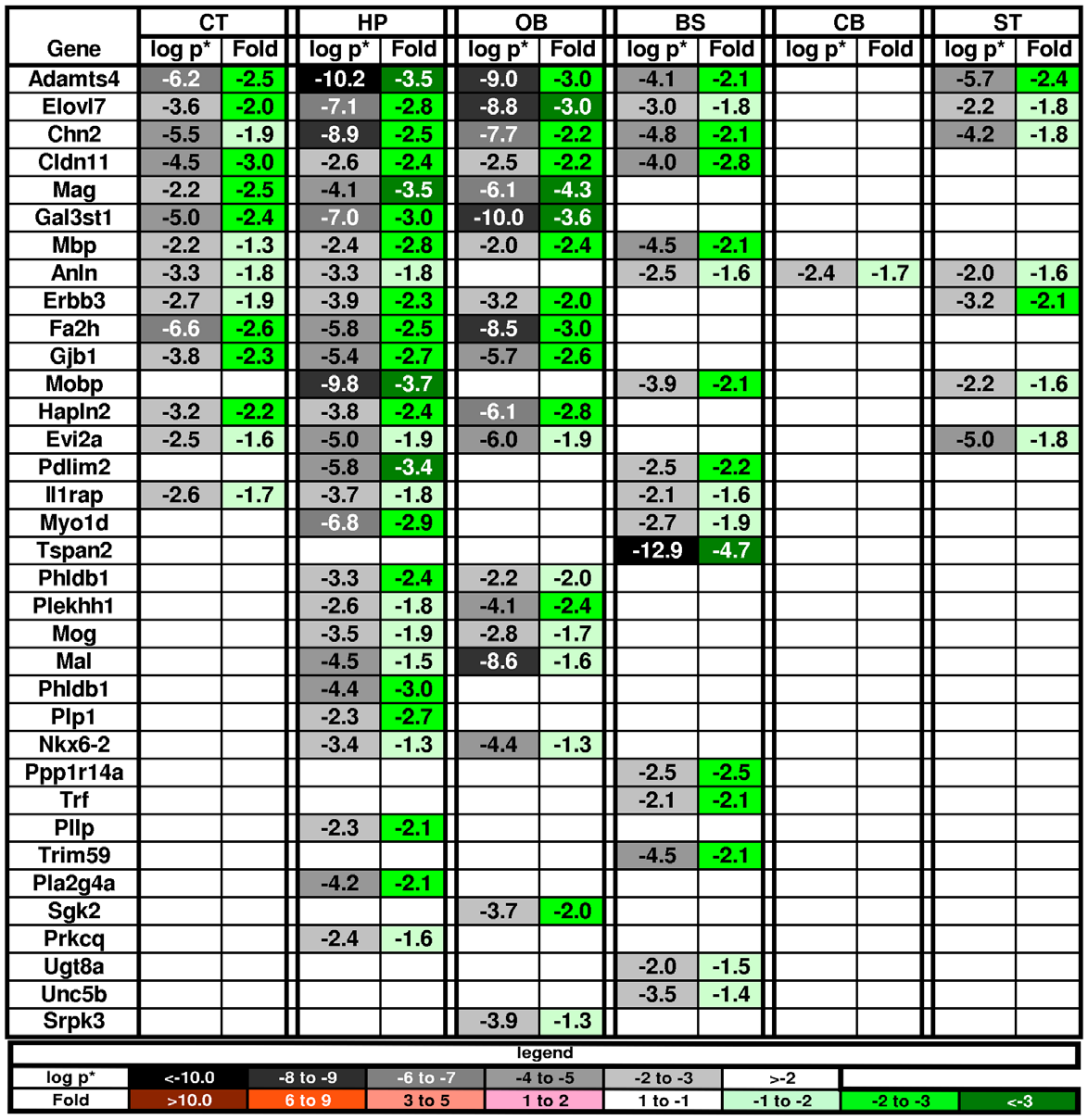
Oligodendrocyte gene changes in the MPS VII brain. The changes were all in the down-regulation direction suggesting dysmyelination. Note the paucity of changes in the cerebellum. Key as in Fig. 2.

Many genes associated with oligodendrocytes were down-regulated even though MPS diseases as a group are not classsified as leukodystrophies. Of the genes identified as oligodendrocyte markers in vitro [32], 53% were changed in the MPS VII brains and they were all down-regulated (Figure 8). The reduced expression of Aspa, Mbp, Mobp, Plp1 and Olig2 were confirmed by RT-PCR (Fig. S1). Interestingly, there were changes in myelin associated genes in all regions except the CB. Many additional genes known to be involved in myelination but not in the list of top marker genes identified in vitro [32], were also altered in the MPS VII brains and most were down-regulated. Ten of the 60 genes from the nervous system development category (see above) are involved in myelination, of which 8 were down-regulated. The 2 that were up-regulated, were only altered in the OB: Ptprz1 (protein tyrosine phosphatase, receptor-type, Z polypeptide 1) is expressed in the remyelinating oligodendrocytes of multiple sclerosis lesions [46]; and Pmp22 (peripheral myelin protein 22) is a component of myelin. Three additional myelin-associated genes (by GO-term) were also up-regulated in the OB: Cd9 (in the CT as well as OB) is associated with late events in myelin maturation [47]; Lgi4 promotes the proliferation and differentiation of glial lineage cells in the peripheral nervous system [48]; and Tgfb1 is induced by activated microglia [49].

#### 5c. Transport and ion channels

Eight functional clusters in the DAVID analysis containing 137 altered genes were associated with solute carriers and ion channels. Changes were found in voltage gated proton, potassium, and chloride channels, but not in sodium or calcium voltage gated channels. Only one changed gene was associated with action potential (Kcnn2) and it was only changed in the BS, which is consistent with the dearth of neuron-associated changes (see above).

#### 5d. Cell adhesion and extracellular matrix

The fourth and seventh highest ranked DAVID functional clusters of genes involved cell adhesion and binding to carbohydrates, polysaccharides and glycosaminoglycans. Three gene changes were seen in all brain regions: Gpnmb (see 4c above) which was highly up-regulated; Clec7a, a C-type lectin, a microglia marker gene that recognizes glucans, which was highly up-regulated [50]; and Selplg, which was down-regulated and is the ligand for P-selectin [51].

#### 5e. Signaling, cell cycle, nucleus, and gene regulation

The DAVID analysis found 5 functional annotation clusters containing 210 genes that thematically grouped into 4 main categories: mapkkk, Egf-like, nucleotide binding, and kinase-related activity. The OB had the largest number of changes, including 3 times as many with p*<10^−7^ as any other region. The total number of genes changed and degree of change was second only to the immunity cluster. Nevertheless, when we examined all the changes in the context of the pathways in which they occurred, none of them showed a crucial junction point or a group of changes within one pathway that would implicate a specific pathway as deficient.

#### 5f. Metabolism

This broad based term involves many different systems, both anabolic and catabolic. Alterations in 3 DAVID functional annotation clusters included 54 genes, with the largest number of changes (30) found in the OB. Most of the changed genes were up-regulated, with only 4 being down-regulated (including Gusb). The top changes were in lysosomal enzyme genes, which are included in this category due to their catabolic function. One interesting change was Aldoc, a glycolytic enzyme that was highly up-regulated (p* = 10^−11^) in all regions except the CB. Aldoc is directly inducible by hypoxia inducing factor 1a [52], which was down-regulated in the CB. Another change limited to the CB was in klotho (Kl), which had the largest fold change in the metabolism genes with an 8-fold over-expression. Klotho is a novel β-glucuronidase potentially capable of hydrolyzing steroid β-glucuronides [53]. The metabolic gene Fa2h (fatty acid 2-hydroxylase) was down-regulated in the CT, HP and OB. Mutations in the Fa2h gene are associated with leukodystrophic dys-myelination and other brain neurodegeneration [54].

#### 5g. Olfactory bulb neuronal changes

Changes specifically attributable to neurons were observed in the OB. Olfactory marker protein (OMP), a gene that is expressed almost exclusively on mature primary olfactory sensory neurons [55], was highly down-regulated (11-fold with p*<10^−13^). Mice that lack OMP have perturbed electrophysiological activity and altered spatiotemporal activity patterns for different odorants [28], [56]. No changes in the olfactory neuronal receptor genes were found suggesting that neuronal loss is not responsible for the OMP decrease. Changes in human olfaction have been shown and often precede diagnosis in a number of immune/inflammatory associated brain diseases such as MS, Alzheimer and Parkinson's disease [57].

## Discussion

Assaying the brain in separate anatomic structures, and using a large cohort of mice for robust power, allowed detection of a large number of specific, highly significant changes in gene expression. The changes shared by multiple regions were mostly attributable to generalized cellular functions (e.g. lysosomal genes) or widespread pathology (e.g. activated microglial genes). However, most changes occurred in only one region, reflecting regional gene specialization, and specific categories of genes varied in number and direction-of-change by region. For example, the OB had the largest number of altered genes, which included some of the most highly changed genes in any region (e.g. OMP, important functions in olfaction). Additionally, the OB had more changes indicative of inflammation than other areas. For oligodendrocyte and myelination genes, the hippocampus had the most changes, whereas the cerebellum had only one, which parallels the magnitude and direction of changes in storage [9].

Some systems were changed primarily in one direction, e.g. most oligodendrocyte genes were down-regulated and most lysosomal genes were up-regulated, while other systems were mixed, e.g. apoptosis. Several of the changed pathways were concordant with known alterations in lysosomal, immunological, astroglial, neurodegenerative, and metabolic systems. Additionally, however, the power and regional analysis were sensitive enough to detect changes in systems involved in neurological dysfunction but which have not been previously appreciated in MPS brains, indicating they are contributing to the overall pathological milieu that results in brain disease.

### Immune and inflammatory system

One of the most interesting categories involved the immune/inflammation system, a general aspect of MPS disorders and neurodegenerative diseases [8], [58], [59], There were notable differences between MPS VII and the closely related MPS IIIb which is the best-studied MPS mouse model for gene expression in the brain [58], [60], [61], [62]. Some of the most highly up-regulated genes in MPS VII were associated with activation of microglia (e.g. Cd68, Mpeg1), as in MPS IIIb, but the MPS VII brain had a notable absence of changes in most genes associated with inflammation, which are significantly changed in MPS IIIb. The few changes in inflammatory genes in MPS VII occurred mostly in the OB. Additionally, lower amounts of A-beta have been found in MPS VII versus MPS IIIb [63]. Consistent with this, MPS IIIb accumulates PHF-Tau in a select region of the cerebral cortex [64], but is not altered in the MPS VII brain [3].

Dissimilar signaling from the predominant, undegraded GAG of each disease may drive the differences in immune and inflammatory phenotypes between MPS VII and MPS IIIb. Substrate specificity of each mutated enzyme results in accumulation of only heparan sulfate (HS) in MPS IIIb whereas MPS VII cells primarily accumulate chondroitin sulfates (CS), with lesser amounts of HS and dermatan sulfate (DS) [8], [65]. In MPS I mice (a model of Hurler disease caused by alpha-L-iduronidase deficiency) both HS and DS accumulate and the mice have similar changes in microglial activation genes as MPS IIIb; however, genes associated with inflammation are not up-regulated (e.g. Ifitm1, Ifnar2 ) [58]. This suggests that the effect of DS on inflammatory and immune gene pathways may be similar to CS, consistent with their closely related structures [66]. These differences may have functional significance because human Sanfilippo disease patients have severe intellectual impairment whereas Sly disease patients are much more variable with some having near normal intelligence [8].

The differences are consistent with known effects of HS and CS on inflammation and the immune system in non-LSD brains [67], [68]. HS stimulates dendritic cells through Tlr4 in MPS IIIb [61] whereas CS has immunomodulatory and anti-inflammatory effects [69], [70], [71]. Also CS disaccharide can induce microglia to adopt a regulatory phenotype that is anti-inflammatory, IFN-gamma counter-regulatory, and neuroprotective [69], [70].

A candidate gene for mediating the diminished inflammatory phenotype of MPS VII is Gpnmb, which was the most significantly up-regulated of all gene changes in MPS VII yet was not altered in MPS I or MPS IIIb. Gpnmb is expressed on antigen presenting cells [72] and blocks the production of inflammatory cytokines [73]. Other anti-inflammatory genes were also highly up-regulated in the MPS VII brain (e.g. Ilt3/Gp49a, FcγrIIb, Tyrobp, Cd300a, Cd300lf, Il10rb). Concordantly, a number of inflammatory-associated genes such as cytokines, chemokines and Cd genes were down-regulated.

### Myelination

The strong down-regulation of oligodendrocyte genes involved in myelination was unexpected since white matter involvement is not seen by histopathology [9]. However, this may be explained by the strong association between neuroinflammation and dysmyelinating diseases in general [59]. Although white matter changes are not observed histopathologically in MPS VII, we recently found that a reduction in white matter can be detected by high-resolution, ex vivo MRI analysis [74]. Thus, the changes in oligodendrocyte genes appear to correlate with mild dysmyelination. This may have a functional consequence because the gene changes that were identified by the GO term “regulation of action potential” are linked to oligodendrocytes rather than neurons.

### Neurodegeneration

Children with MPS VII show varying amounts of mental retardation [8], but there were no changes in the neuronal marker genes [32]. Moreover, of 159 probes for 8 classes of neuron receptors, only four changes were found, all in different regions and all with low significance. In contrast, neurodegeneration lesions are found in the HP, ST and CT of MPS VII mice [3]. They are characterized by excess ubiquitin and neurofiliment accumulation and up-regulation of the ubiquitin specific peptidase 53 (Usp53) gene was detected in the HP, OB, BS and ST. The ubiquitin-proteosome system (UPS) is implicated in the pathogenesis of several neurodegenerative disorders [75] and is also important in neuroprotection [76].

### Developmental pathways

Many changes were seen in genes associated with neural development and differentiation. However, no specific pathway or subset of developmental processes was implicated, suggesting a more diffuse effect resulting in activation of different gene sets in different cell types. The major elements of brain anatomical patterning are maintained in the MPS VII brain and there is no overt cell loss [9], despite the presence of a neurodegenerative phenotype [3]. Thus the activation of developmental genes may be triggered by the diseased microenvironment. CS affects several aspects of nervous system development directly [77]. In addition, the CS-rich microenvironment of the diseased brain affects differentiation of neural stem cells in the MPS VII dog [78]. The changes in developmental genes may also be from reactive astrocytes, which exhibit NSC characteristics [79] as MPS VII mice have diffuse astrogliosis [3]. Two major markers of reactive astrocytes were up-regulated: Gfap in all regions and vimentin (Vim) in CT, OB, and ST.

A substantial number of the altered developmental and differentiation genes are associated with non-CNS tissues, many with the skeletal system. During embryogenesis the neural crest is associated with development of both the nervous and skeletal systems [80], [81], suggesting that fundamental developmental programs may be reactivated under the disease conditions.

### Summary

The robust power of this study revealed a large number of gene expression changes and variability by major brain subregion. Some of the differences between the normal and MPS VII brains were concordant with known histopathological, biochemical, and behavioral abnormalities in the MPS VII mouse; but, a number of altered systems have not been implicated previously and provide fruitful directions for further investigation. The central role of CS in signaling processes, cell-cell interactions, maintenance of the extracellular space, and development [77] may account for many of the gene alterations secondary to the primary monogenic mutation. The differences in patterns of expression between the immune and inflammatory system genes in MPS VII compared to MPS IIIb appear to directly reflect the predominant GAG accumulated in each mutation. The decrease in expression of major oligodendrocyte genes indicates defects in myelination. The perturbations in developmental genes, while not implicating any single process, is consistent with the apparent delayed developmental potential of neural progenitor cells due to the microenvironment, which would be expected to be pleiotropic. The large number of gene changes in the OB and the availability of gene vector and neural stem cell reagents for manipulating the OB in the MPS VII model [82], [83], indicate that olfaction may be useful for probing the mechanisms of CS accumulation on a sensory perception system in the brain.

These data also provide a basis to investigate the differences between changes in gene expression and their manifestation as protein changes and effects on cell function. Although further understanding of the pathological effects will require specific experiments targeted to the biochemistry and physiology of each pathway, understanding which changes lead to specific pathological effects should result in a better understanding of the relative contributions of the myriad altered sub-systems to overall neuropathology. Furthermore, better understanding of the multitude of effects in the diseased brain will provide more specific parameters to evaluate the effect of therapies.

## Materials and Methods

### Ethics statement

All animal procedures were performed according to protocols approved by the IACUC (Institutional Animal Care and Use Committee) of the Children's Hospital of Philadelphia (CHOP).

### Online Data Repository

The primary microarray data for this paper is available at http://www.ncbi.nlm.nih.gov/geo/ accession number GSE34071.

### MPS VII animal model

Wild-type (*gus^+^/gus^+^*), carriers (*gus^mps^/gus^+^*) and MPS VII-affected (*gus^mps^/gus^mps^*) on the C3H-HeOuJ background [84] were maintained in our breeding colony through carrier-carrier brother-sister mating. Identification of the MPS VII-affected mice, which contain a single base pair deletion in exon 10 of the GUSB gene, was verified by PCR genotyping, as described previously [85]. Diseased and normal mice were housed together until brain harvest at 5 months of age. Four litters were used that were born within one week of each other and each litter contained at least one mutant and one normal (genotypic carrier or wild-type) pup. The group included 3 genotypic normal (Wt), 5 carrier (C) and 8 MPS VII (M) mice. To obtain a sufficient number of paired animals for the study, it was necessary to use a mixture of genders for both the normal control group (2 female, 6 male) and MPS VII group (4 female, 4 male). Thus the gene expression data from males and female were considered as one group of normal (Wt/C) or MPS VII (M) brains.

### Micro-dissection of brains

At 21–22 weeks of age mice were anesthetized with ketamine/xylazine and the brains were removed and placed immediately on ice. The hemispheres were separated along the medial longitudinal fissure and each hemisphere was further dissected based on anatomical boundaries to obtain the following brain regions: cerebellum (CB), brainstem (BS), olfactory bulb (OB), cerebral cortex (CT), hippocampus (HP), and the remaining mid and forebrain portion which included the striatum and thalamus (ST). The brain pieces were immediately frozen in liquid nitrogen and stored at −80C° until used for RNA isolation.

### RNA isolation

Total RNA was isolated separately from each of the six regions of the right hemisphere. Frozen regions were placed into TRIzol (Invitrogen) at 1 ml per sample and homogenized (Pellet Pestle Motor - Kontes, VWR) at maximum speed for 20–40 Sec. The RNA was further purified using the RNeasy Lipid Tissue mini kit (Qiagen) according to manufacturer's instructions. Total RNA quality was assessed by measuring the A_260/280_ ratio on a NanoDrop ND-1000 spectrophotometer (Thermo Scientific). RNA integrity was verified by visualization of the 28S and 18S ribosomal rRNA bands, with no presence of smear, using a denaturing TAE- agarose gel.

### Microarrays

1 µg RNA from each brain region was used to prepare biotinylated aRNA samples using the MessageAMP II-biotin Enhanced Signal Round aRNA Amplification Kit (Ambion). Reverse transcription, in vitro transcription and fragmentation were performed according to manufacturer's instructions (Ambion). Samples of 10 µg aRNA were hybridized on Affymetrix mouse genome 430A 2.0 Gene Chips containing 22,690 oligonucleotide probe sets (www.affymetrix.com). A total of 96 samples (16 brains, 6 regions each) were hybridized. Hybridization, staining and washing were performed on an Affymetrix Fluidics Station 400 at the Children's Hospital of Philadelphia Nucleic Acid Core facility according to Affymetrix protocols. Scanning was performed using the Affymetrix Gene Chip Scanner 3000 controlled by a GeneChip Operating software 1.4 (GCOS, Affymetrix).

### Data normalization and analysis

Raw microarray image files were processed using the Affymetrix GCOS 1.4 software to calculate individual probe cell intensity data and generate CEL data files. The 96 Affymetrix CEL files were imported into Partek Genomics Suite (v6.5, Partek Inc. St. Louis, MO) where GCRMA was applied. Clustering approaches and principle components analysis showed two of the arrays were outliers, which were excluded for subsequent statistical analysis.

For the remaining 94 arrays, a 3-way mixed model ANOVA was calculated including the factors genotype (2 levels with wild-type and carrier combined as “normal” as described above), region (6 levels), and mouse ID (16 levels). An interaction term between genotype and region was included as well. In conjunction with the ANOVA, we calculated pairwise contrasts (each yielding a p-value and a fold change) for genotype for each of the 6 brain regions. For all resulting p-values, a corrected step-up p-value (p*) (False Discovery Rate by the Benjamini Hochberg step-up method as implemented in Partek) was calculated. A p*≤0.01 without prejudice for fold change was used to select the genes differentially expressed in MPS VII mice.

For regional gene enrichment in the normal mouse, a two way mixed model ANOVA was calculated using the factors region (6 levels) and mouse ID (8 levels). p* was calculated as above. Regional gene enrichment fold in the normal brain was calculated by dividing the regional average by the average of all the remaining regions.

### Functional analysis of the data sets

The significantly changed (p<0.01) genes were analyzed using GeneTrail (http://genetrail.bioinf.uni-sb.de/) [17] for Gene Ontology (GO) term [18] and KEGG [19] pathway enrichment, DAVID (http://david.abcc.ncifcrf.gov) [20] for GO-term and other database functional clustering, or from literature-search generated gene lists as described in the results section. Hierarchical clustering of the differentially expressed genes and heatmap generation was carried out using the MultiExperiment Viewer (MeV) tool in TM4 Microarray Software suite (http://www.tm4.org/mev.html) [86].

### Quantitative RT (reverse transcriptase)-PCR and data analysis

To validate the microarray data, nine differentially expressed genes were chosen for quantitative real time PCR analysis (Q-PCR) using the following TaqMan gene expression assays: beta-glucuronidase (Gusb, Mm03003537_s1); hexosaminidase B (Hexb, Mm00599880_m1); glial fibrillary acidic protein (Gfap, Mm00546086_m1); lysozyme (Lyz2, Mm00727183_s1), myelin-associated oligodendrocytic basic protein (Mobp, Mm00485088_m1), myelin basic protein (Mbp, Mm01262035_m1), proteolipid protein (myelin) 1 (Plp, Mm00456892_m1), oligodendrocyte transcription factor 2 (Olig2, Mm01210556_m1), and aspartoacylase (aminoacylase) 2 (Aspa, Mm00480867_m1). The 18 s ribosomal subunit (18 s rRNA, Hs 99999901_s1) was used as a reference gene.

The same RNA samples used in the microarray analysis were also used for Q-PCR. The total RNA samples treated with DNase (turbo DNA free, Ambion) and then reverse transcribed using a high capacity cDNA reverse transcription kit (Applied Biosystems) and then amplified. The changes in reporter fluorescence were monitored using the ABI SDS 7500 system (Applied Biosystems) run with the standard program. The data is expressed as mean fold changes in the MPS VII compared to the normals. Similar results in both the direction and the magnitude of change were achieved by both assay methods (Fig. S1).

### Rationale for grouping wild-type and carriers together

The disease requires inheritance of homozygous mutant alleles and there is no evidence of disease in heterozygous carriers in mice or humans. An initial analysis on HP samples using hierarchical clustering of the expression values of the 50 top and bottom significantly altered genes by fold change (p<0.01) between Wt, C and M mice, clustered the wild-type and carrier mice together and distinct from the mutants (Fig. S2). Also, the larger microarray dataset was compared using a principal component analysis (PCA) derived from an ANOVA, which compared genotype, region, and the interaction between the them for the most significant 500 probes, which showed that the wild-type and carrier samples segregated together and the mutants segregated separately (Fig. S3). This genotypic analysis is consistent with the known phenotype in which carriers and normals are indistinguishable [21], [84]. Thus for analysis, we grouped the genotypically homozygous wild-type (Wt) and heterozygous carrier (C) samples together in a group that has a normal phenotype (N).

### Data presentation for figures

When multiple probes are present for the same gene, the probe data with less significance has been eliminated for brevity. If the eliminated probe showed statistically significant regional information not found in the non-eliminated probe data, the data was consolidated for the purpose of presentation.

## Supporting Information

Figure S1 **Comparison of fold change by RT-PCR and microarray for selected genes.**
(TIF)

Figure S2 **Heatmap showing that the normal and carrier groups are similar to each other and distinct from the mutants.** The top and bottom 50 hippocampal genes are displayed; the values are the difference from the mean.
(TIF)

Figure S3 **Plot of Primary Component Analysis (PCA) by genotype and region showing that carriers and wild-type samples segregate from mutants.**
(TIF)

Table S1 **Thematic consolidation of DAVID annotation clusters.**
(XLS)

Table S2 **Gene expression altered or unaltered exclusively in one region (orange = upregulated/green = downregulated).**
(XLS)

Table S3 **Neuronal receptor changes.**
(XLS)

Table S4 **Gene expression changes associated with the GO terms Positive regulation of apoptosis and Negative regulation of apoptosis (orange = upregulated/green = downregulated).**
(XLS)

Table S5 **Changes in astrocyte marker genes.**
(XLS)
