# Supplementary figures and images for: Dysregulation of Gene Expression in a Lysosomal Storage Disease Varies between Brain Regions Implicating Unexpected Mechanisms of Neuropathology
Source: PLoS One. 2012 Mar 5;7(3):e32419. doi: 10.1371/journal.pone.0032419 (PMC3293807; doi:10.1371/journal.pone.0032419)

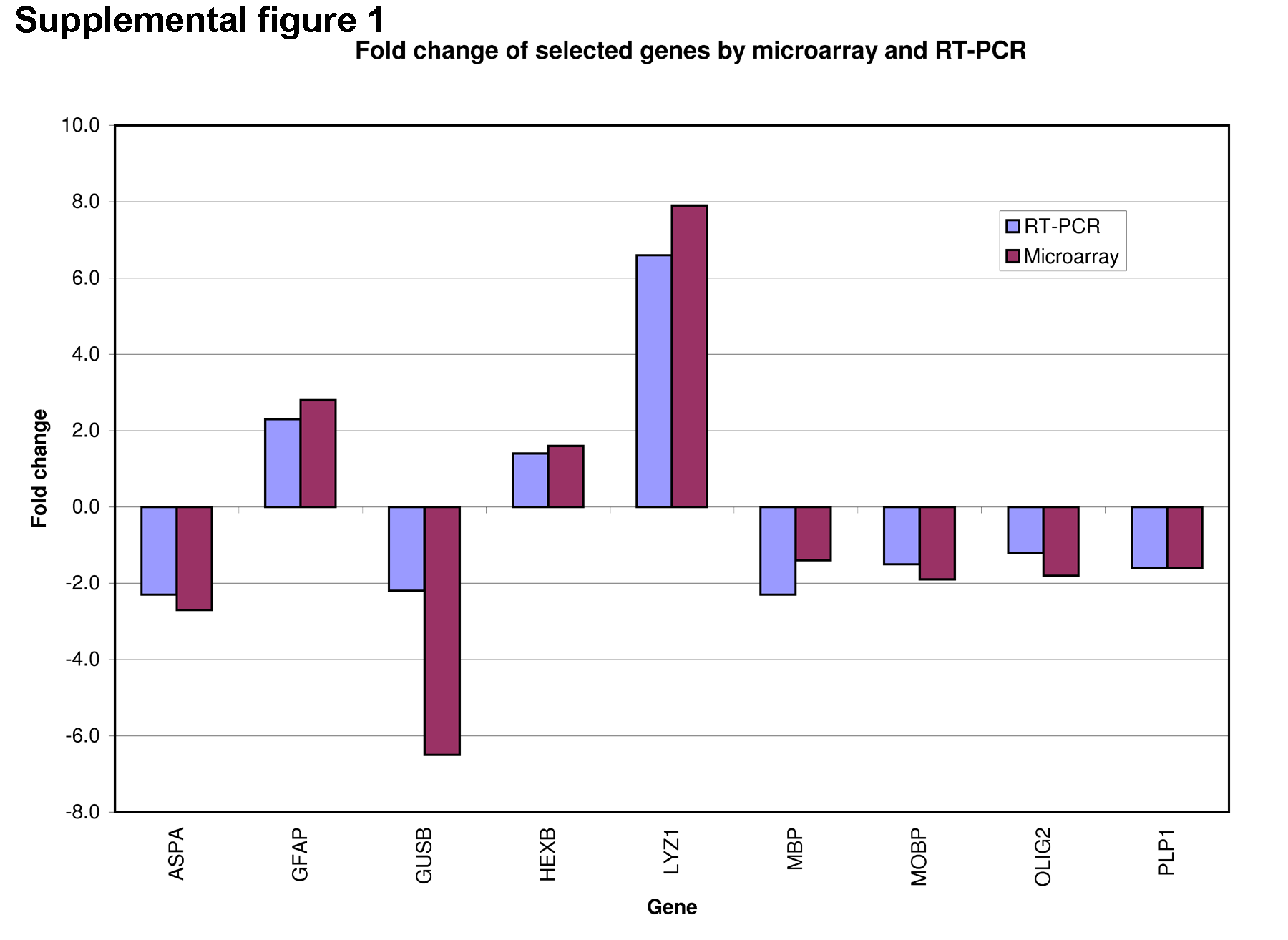

Supplement: Figure S1 — Comparison of fold change by RT-PCR and microarray for selected genes. (TIF) [file pone.0032419.s001.tif]

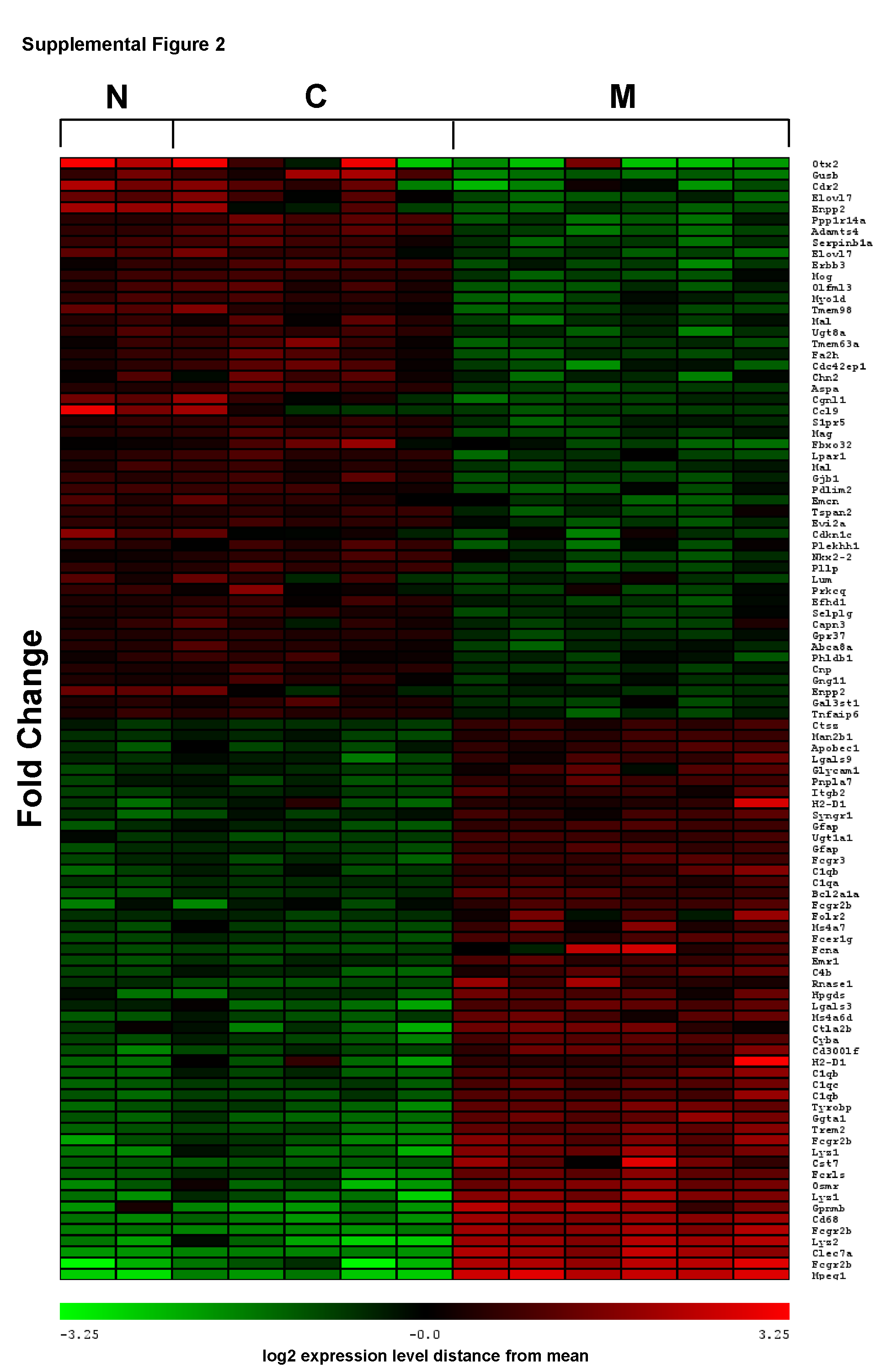

Supplement: Figure S2 — Heatmap showing that the normal and carrier groups are similar to each other and distinct from the mutants. The top and bottom 50 hippocampal genes are displayed; the values are the difference from the mean. (TIF) [file pone.0032419.s002.tif]

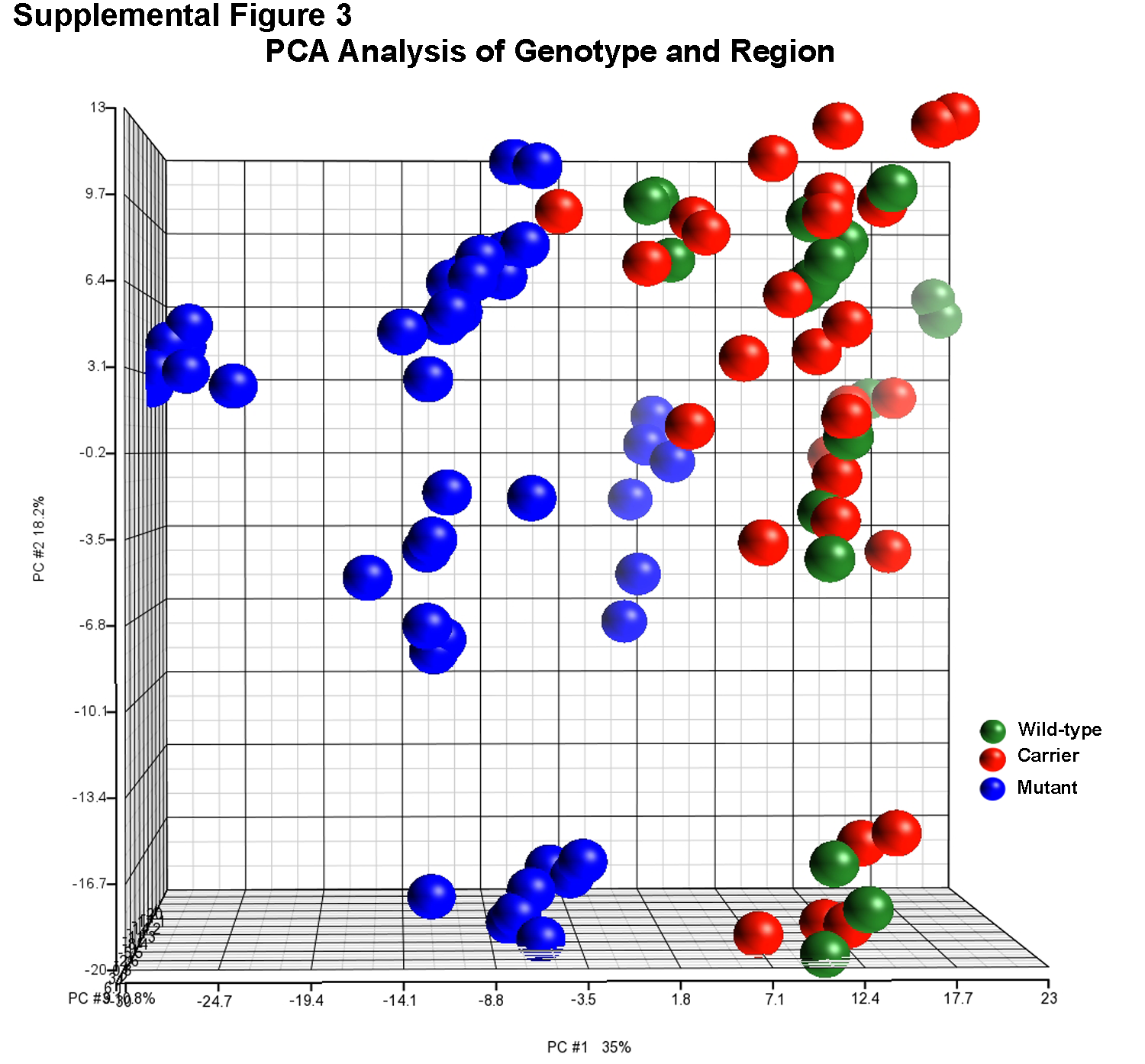

Supplement: Figure S3 — Plot of Primary Component Analysis (PCA) by genotype and region showing that carriers and wild-type samples segregate from mutants. (TIF) [file pone.0032419.s003.tif]
